# Gastric Antral Perforation Caused by a Percutaneous Endoscopic Gastrojejunostomy Tube: A Case Report

**DOI:** 10.70352/scrj.cr.26-0140

**Published:** 2026-05-02

**Authors:** Toru Zuiki, Takashi Ui, Jun Oki, Fumi Sakatsume, Kaori Nakajima, Imiko Iwami, Fumie Ogawa, Aya Numata, Natsuki Sakayori, Miyuki Tajima

**Affiliations:** 1 Department of Surgery, Yuki Hospital, Yuki, Ibaraki, Japan; 2 Department of Nutrition, Yuki Hospital, Yuki, Ibaraki, Japan; 3 Department of Pharmacy, Yuki Hospital, Yuki, Ibaraki, Japan; 4 Department of Rehabilitation, Yuki Hospital, Yuki, Ibaraki, Japan

**Keywords:** percutaneous endoscopic gastrostomy, percutaneous endoscopic gastrojejunostomy, PEG-J, gastric perforation, enteral nutrition, complication

## Abstract

**INTRODUCTION:**

Percutaneous endoscopic gastrojejunostomy (PEG-J) is used when gastric feeding via percutaneous endoscopic gastrostomy (PEG) is poorly tolerated due to gastroesophageal reflux or delayed gastric emptying. Gastrointestinal perforation related to PEG-J has been reported mainly in infants; gastric perforation in adults is extremely rare. We report a case of gastric antral perforation caused by the tube tip 12 days after exchange from a PEG tube to a PEG-J tube.

**CASE PRESENTATION:**

A 49-year-old man with severe dysphagia after brainstem hemorrhage underwent PEG placement using the introducer (direct puncture) technique, and a low-profile button was inserted into the anterior wall of the mid-gastric body. During 1 month of gastric feeding, he developed recurrent nausea and vomiting. Endoscopy showed mild antral torsion without obstruction, and reflux was considered the primary cause. The tube was exchanged for a double-lumen PEG-J tube under endoscopic guidance; however, the jejunal extension could not be advanced beyond the second portion of the duodenum and showed intragastric looping, so the tube was secured with residual intragastric looping and the tip was positioned in the second portion of the duodenum. Enteral feeding was initially tolerated. On day 12, he developed acute right-sided abdominal pain, and CT demonstrated extraluminal migration of the tube tip. Emergency laparotomy revealed perforation of the anterior wall of the greater curvature side of the gastric antrum, just proximal to the pylorus, with localized peritonitis and limited contamination. The perforation was closed primarily and reinforced with an omental patch. To secure a route for enteral nutrition, a gastrojejunostomy with an additional Braun enteroenterostomy was performed. The postoperative course was uneventful, and enteral nutrition was resumed without recurrent reflux symptoms.

**CONCLUSIONS:**

Intragastric looping and tube rigidity may cause persistent, focal pressure on the gastric wall and lead to perforation. Careful attention to tube configuration, appropriate device selection, and prompt evaluation of abdominal pain after PEG-J placement are essential.

## Abbreviations

Fr
: French
PEG
: percutaneous endoscopic gastrostomy
PEG-J
: percutaneous endoscopic gastrojejunostomy

## INTRODUCTION

PEG was first described by Gauderer et al. in 1980,^1)^ and has since become widely used for enteral nutrition in patients with dysphagia due to various etiologies. Several PEG techniques are available, including the pull, push, and introducer methods^2)^; however, the fundamental concept is the same: percutaneous placement of a feeding tube into the stomach. At our institution, we commonly use a 1-step direct puncture technique after gastropexy, followed by placement of a low-profile button-type gastrostomy tube.

In patients who experience frequent vomiting due to gastroesophageal reflux after PEG placement, we often exchange the gastrostomy tube for a transgastric jejunal feeding tube (percutaneous endoscopic gastrojejunostomy [PEG-J]) to reduce aspiration risk. Because PEG-J tubes are typically double-lumen devices, gastric decompression and jejunal feeding can be achieved simultaneously.^3)^

Herein, we present a case of gastric antral perforation caused by a PEG-J tube 12 days after tube exchange, and discuss the presumed mechanism and preventive measures. Reports of gastrointestinal perforation associated with PEG-J are rare, and to our knowledge, this is the first reported case of the tube tip perforating the stomach wall.

## CASE PRESENTATION

A 49-year-old man was transferred from a rehabilitation hospital for PEG placement and subsequent rehabilitation because of severe dysphagia following a brainstem hemorrhage. Although his swallowing dysfunction was profound, he was able to produce limited speech and communicate simple intentions. Preoperative abdominal CT showed no abnormal findings. Upper gastrointestinal endoscopy demonstrated no gastric mucosal atrophy and only mild reflux esophagitis.

**Fig. 1:**
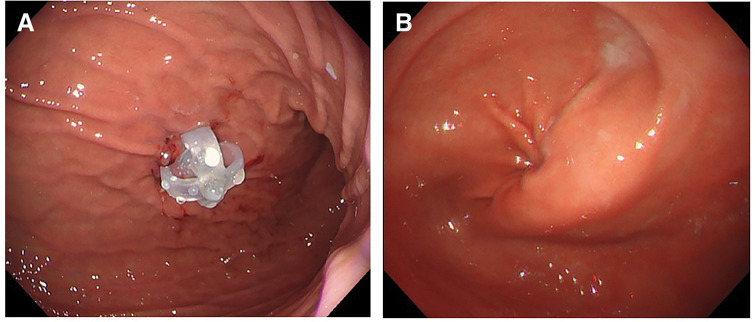
(**A**) PEG was performed using a direct puncture technique after gastropexy, and a 24-Fr low-profile button gastrostomy tube (shaft length 3.5 cm) was placed on the anterior wall of the lower-to-mid gastric body. (**B**) Endoscopy performed for reflux symptoms showed mild torsion of the gastric antrum. There was no stenosis, and passage of a slim endoscope was possible. Fr, French; PEG, percutaneous endoscopic gastrostomy

PEG was performed using a direct puncture (introducer) technique with 3-point gastropexy, and a low-profile button gastrostomy tube (24 Fr; shaft length 3.5 cm; Ideal Button, Olympus Medical Systems, Tokyo, Japan) was inserted into the anterior wall of the lower/mid gastric body (**Fig. 1A**). Enteral feeding was initiated the following day.

After approximately 1 month of gastric feeding via PEG, the patient frequently complained of nausea and vomiting. Endoscopy showed mild reflux esophagitis but no obstructive lesions that could explain vomiting. Mild antral torsion was noted (**Fig. 1B**), making direct visualization of the pyloric ring slightly difficult; however, passage of a slim endoscope was not impaired. We therefore decided to exchange the gastrostomy tube for a PEG-J tube.

**Fig. 2:**
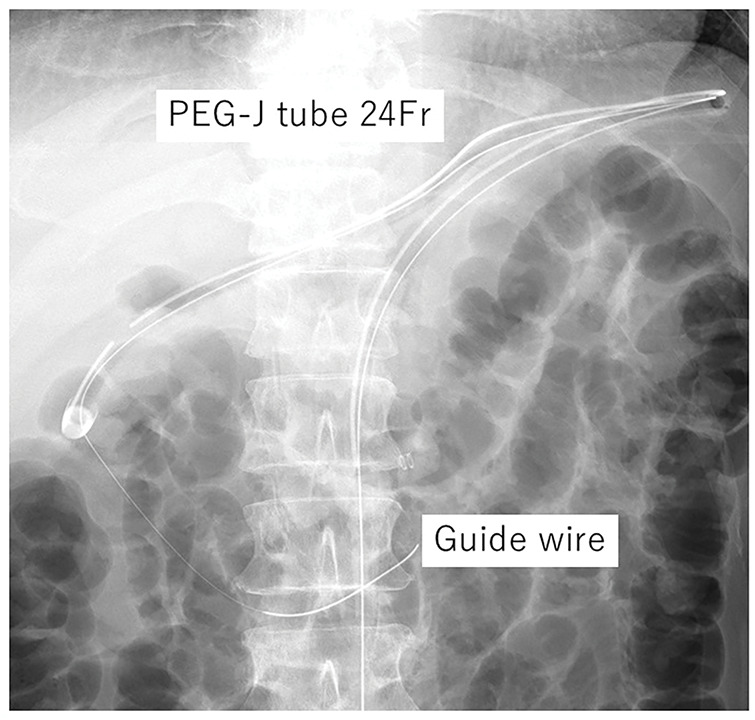
A slim endoscope was inserted through the gastrostomy, and a guidewire was advanced beyond the ligament of Treitz into the jejunum. A 24-Fr PEG-J tube was introduced; however, it could not be advanced beyond the second portion of the duodenum. The tube was fixed with residual intragastric looping, with the tip left in the second portion of the duodenum. Fr, French; PEG-J, percutaneous endoscopic gastrojejunostomy

A slim endoscope was advanced via the gastrostomy tract to the third portion of the duodenum. A guidewire was placed beyond the ligament of Treitz. Although a 24-Fr double-lumen gastrojejunostomy tube (GB Jejunal Button, Fuji Systems, Tokyo, Japan) was introduced over the wire, the distal segment could not be advanced beyond the second portion of the duodenum, repeatedly resulting in intragastric looping of the jejunal limb. Despite additional maneuvers (hydrophilic guidewire exchange and transoral endoscopic guidance), deep advancement was unsuccessful. The tube was therefore secured at the gastrostomy site despite residual intragastric looping, and the tip was left in the second portion of the duodenum (**Fig. 2**). Although we expected peristalsis to advance the tube distally, plain radiography obtained 3 days later showed no further advancement of the tip. Nevertheless, jejunal feeding was tolerated without symptoms, and gastric decompression through the gastric side holes was not required.

**Fig. 3:**
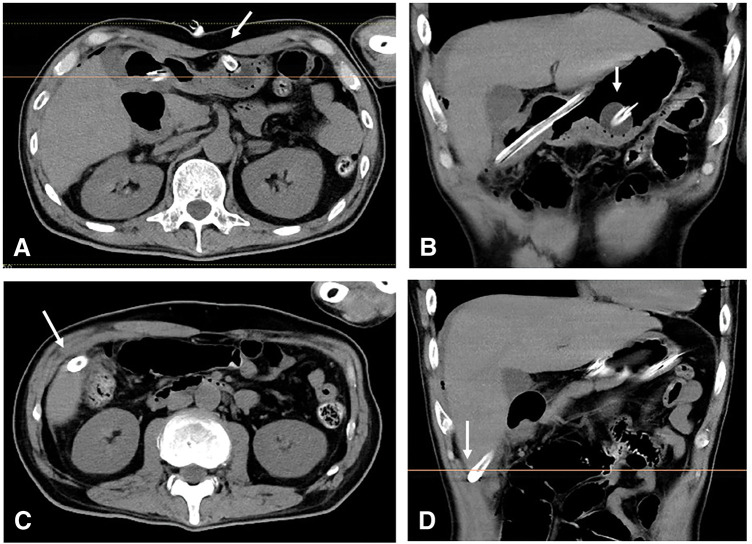
CT performed for acute right-sided abdominal pain on day 12 after PEG-J exchange. (**A**) Gastrostomy site was fixed at the gastric body (arrow). (**B**) Gastrojejunostomy tube was secured with a balloon (arrow). (**C**) Gastrojejunostomy tube was located beneath the liver (arrow). (**D**) The tube tip was located outside the intestinal lumen (arrow). PEG-J, percutaneous endoscopic gastrojejunostomy

On the night of day 12 after the PEG-J exchange, the patient developed acute right-sided abdominal pain. Feeding was stopped immediately, and CT demonstrated that the PEG-J tube tip had migrated outside the intestinal lumen and was located beneath the liver (**Fig. 3**).

**Fig. 4:**
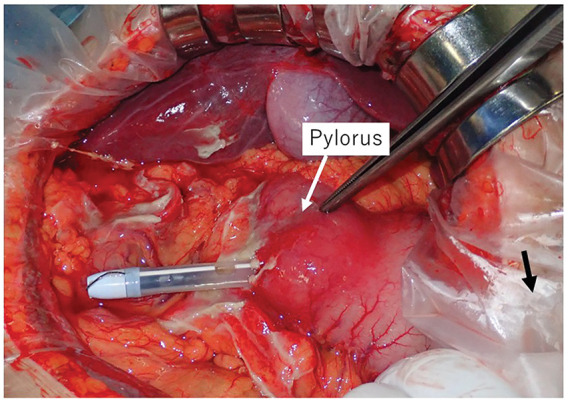
Emergency laparotomy revealed gastric antral perforation caused by the PEG-J tube at the greater curvature of the gastric antrum. Peritoneal contamination was mild. The gastrostomy site was covered with a wound retractor and could not be visualized (black arrow). PEG-J, percutaneous endoscopic gastrojejunostomy

**Fig. 5:**
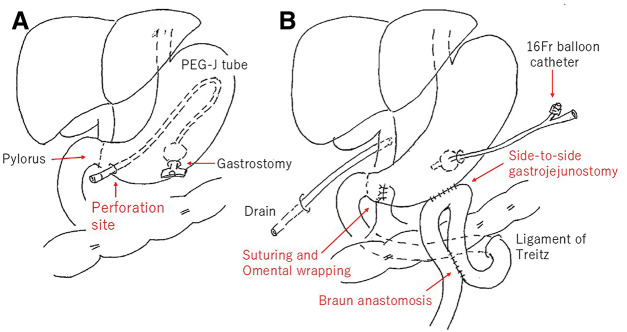
(**A**) Intraoperative findings demonstrating the perforation site and the PEG-J tube. (**B**) The gastric perforation was closed with primary sutures and covered with an omental patch. A gastrojejunostomy bypass with an additional Braun enteroenterostomy (jejunojejunostomy) was performed, and the gastrostomy was maintained with a balloon catheter. Fr, French; PEG-J, percutaneous endoscopic gastrojejunostomy

Emergency laparotomy revealed that the PEG-J tube had perforated the anterior wall of the gastric antrum of the greater curvature, just proximal to the pylorus (**Fig. 4**). Localized peritonitis was present; however, no bile-stained fluid was observed, and contamination was limited (**Fig. 5A**). Because there was minimal crush injury around the perforation site, the defect was closed primarily with sutures and reinforced with an omental patch. The original gastrostomy site was firmly fixed to the abdominal wall, and no abnormalities were observed around it. Antral torsion could not be appreciated from the serosal side.

To secure a route for continued enteral nutrition via the gastrostomy, a gastrojejunostomy bypass was created, and a Braun enteroenterostomy (jejunojejunostomy) was added (**Fig. 5B**). The gastrostomy tract was maintained intraoperatively using a balloon catheter and was exchanged for a balloon-retained button gastrostomy device on POD 3. The postoperative course was uneventful. Enteral nutrition via the gastrostomy was resumed on POD 5, and the patient remained free from vomiting, reflux symptoms, and abdominal pain.

## DISCUSSION

Common indications for PEG-J include recurrent aspiration pneumonia due to gastroesophageal reflux after PEG placement, gastric stasis, and enteral nutrition in patients with pancreatitis or malignancy.^3,4)^ PEG-J is also used for continuous infusion therapy with levodopa–carbidopa intestinal gel in advanced Parkinson’s disease.^5)^ Double-lumen gastrojejunostomy tubes have been applied in various clinical settings by leveraging simultaneous jejunal feeding and gastric decompression. Reported applications include superior mesenteric artery syndrome, pyloric stenosis due to advanced gastric cancer, afferent loop syndrome after total gastrectomy,^6)^ and a colo-duodenal fistula associated with Crohn’s disease.^7)^

In the present case, the most likely contributing factor to gastric perforation was fixation of the PEG-J tube with residual intragastric looping, which is generally undesirable. As an upstream factor, fixation of the gastric body to the abdominal wall after PEG may have predisposed the antrum to torsion, making distal advancement of the jejunal extension tube difficult. Moreover, because of the antral torsion, a partially retrograde tube tip may have been unable to pass through the pylorus despite gastric peristalsis; consequently, the relatively stiff distal portion may have exerted persistent focal pressure on the antral wall, eventually resulting in perforation. While fixing the stomach wall to the abdominal wall via gastrostomy has been reported to be effective in treating gastric volvulus and upside-down stomach,^8)^ there have also been reports of gastric volvulus caused by gastrostomy.^9)^ This appears to be corroborating evidence that a slight twist occurred in the gastric antrum as a result of the gastrostomy procedure in this case.

Commercially available double-lumen tubes range from 16 to 24 Fr; we selected a 24-Fr tube to maintain long-term patency. However, larger-diameter tubes are typically more rigid, increasing the pressure exerted at the tip and making advancement more difficult because of greater weight and frictional resistance. Plastic nasogastric tubes may become more rigid after exposure to gastrointestinal secretions and can cause pressure ischemia.^10)^

A literature search using the terms “PEG-J,” “perforation,” and “complication” suggests that perforation related to gastrojejunostomy tubes is reported more frequently in infants.^11,12)^ A systematic review estimated an overall perforation rate of 2.1% among 2726 pediatric PEG-J placements, with a higher risk in children weighing <10 kg.^12)^ In adults, duodenal bulb perforation due to retrograde migration of the tube tip has been reported.^13)^ A case series of 3 cases of proximal jejunal perforation caused by the tip of a PEG-J extension tube has also been reported.^14)^ In addition, 2 cases of gastric perforation secondary to buried bumper syndrome during PEG-J feeding for necrotizing pancreatitis have been described.^15)^ To our knowledge, an adult case in which the tube tip penetrated the gastric wall after PEG-J placement has not been previously reported (**Table 1**).

**Table 1:** Reported cases of PEG-J–related gastrointestinal perforation in adults

Author (publish)	Patient’s age (years), sex	Perforation site	Mechanism of perforation	Tube size, tube tip	Time from PEG-J placement to perforation (days)
Kalkan et al.^13)^ (2012)	63, male	Duodenal bulb	Migration of the jejunal extension tube	20-Fr PEG with 9-Fr jejunal extension	6
Rosenberger et al.^14)^ (2012)	42, male	Jejunum	Unknown	24-Fr PEG with 12-Fr jejunal extension	12
	58, male				23
	29, male				19
Holt and Varadarajulu^15)^ (2016)	48, male	Gastric body	Bumper migration	Unknown	7
	43, male				4
Present case	49, male	Gastric antrum	Pressure of the tube tip	24-Fr, double-lumen tube, relatively rigid tip	12

Fr, French; PEG, percutaneous endoscopic gastrostomy; PEG-J, percutaneous endoscopic gastrojejunostomy

Based on this experience, we emphasize the following preventive considerations. First, the PEG-J tube should be placed as straight as possible without intragastric looping, because slack can promote retrograde tip migration and flipping. Second, device selection should be individualized: although larger tubes may offer improved patency, increased stiffness may raise the risk of pressure injury and perforation and may hinder distal advancement. A softer, atraumatic tip design may be preferable; in pediatrics, a practice change to soft-tip tubes has been associated with a reduction in perforation events.^16)^ Third, abdominal pain during enteral feeding should be taken seriously. Although perforation in our patient occurred on day 12, perforation in low-birth-weight infants has been reported within 3 days of placement,^11)^ indicating that perforation is not limited to long-term indwelling complications. Clinicians should remain vigilant for ongoing pressure injury by the tube tip and carefully monitor abdominal findings and vital signs.

Beyond perforation, PEG-J–related complications reported in case studies include tube dysfunction (e.g., obstruction), buried bumper syndrome,^5,15)^ intussusception,^17,18)^ small-bowel volvulus,^19)^ and tube migration.^20)^ Although the true incidence of these events is unclear due to the limited number of reports, clinicians should recognize the diversity of potential complications.

If deep advancement is unsuccessful, several options may be considered: (1) switching to a smaller-diameter device to improve maneuverability; (2) switching from a double-lumen PEG-J tube to a PEG-J extension system, balancing the higher risk of clogging with smaller extension sizes and acknowledging that extension-related jejunal perforation has been reported^14)^; (3) abandoning gastric decompression and using a single-lumen jejunal feeding tube; and (4) if a tube must be left with intragastric looping, reattempting distal advancement within a few days using an alternative device or technique. It should be noted that there has been a reported case of a boy in whom the jejunostomy tube perforated the jejunum the day after being switched from PEG to a jejunostomy tube.^21)^

The clinical course of this case was previously presented at the 51st Annual Meeting of the Japanese Society for Parenteral and Enteral Nutrition.

## CONCLUSIONS

This report describes a rare case in which the tip of a PEG-J tube perforated the stomach, along with a discussion of its causes and management. PEG-J can be effective for patients who develop reflux or intolerance during gastric feeding after PEG and has broad clinical applications. However, PEG-J placement requires careful attention to tube configuration, and appropriate selection of tube diameter, functional length, and tip design according to the patient’s anatomy and clinical context. Clinicians should also recognize gastrointestinal perforation as a possible complication and closely monitor symptoms and physical findings after placement, with periodic confirmation of tube position and course when appropriate.

## DECLARATIONS

### Availability of data and materials

The datasets supporting the conclusions of this article are included within the article.
